# Targeting TLR4 Attenuates Endometriosis Progression by Suppressing NF-κB/NLRP3 Inflammasome Activation and Angiogenesis

**DOI:** 10.3390/ijms27094151

**Published:** 2026-05-06

**Authors:** Yunlei Cao, Xiangxiang Zhu, Xinxin Hou, Ding Ding

**Affiliations:** 1 Department of General Gynecology, Obstetrics & Gynecology Hospital of Fudan University, Shanghai Key Lab of Reproduction and Development, Shanghai Key Lab of Female Reproductive Endocrine Related Diseases, Shanghai 200433, China; cyl_fd@126.com; 2 School of Integrative Medicine, Shanghai University of Traditional Chinese Medicine, Shanghai 201203, China; zhuxiangxiang1202@163.com

**Keywords:** endometriosis, toll-like receptor 4, NLRP3 inflammasome, angiogenesis, TAK-242, IL-1β

## Abstract

Endometriosis is a chronic inflammatory disorder affecting approximately 10% of reproductive-age women, yet non-hormonal therapeutic options remain limited. This study investigates the role of the TLR4/NF-κB/NLRP3 inflammasome axis in endometriosis pathogenesis and evaluates the therapeutic potential of pharmacologic TLR4 inhibition. Ectopic endometriotic tissues, eutopic endometrium, and peritoneal fluid were collected from 15 patients with ovarian endometriosis and 15 control subjects. The endometriotic epithelial cell line 11Z was stimulated with LPS and ATP with or without the TLR4 inhibitor TAK-242. A murine endometriosis model was established in wild-type C57BL/6 and TLR4^−^/^−^ mice treated with TAK-242. Expression of TLR4, p-p65, NLRP3, caspase-1, cleaved caspase-1 (p20), GSDMD-N, IL-1β, PCNA, and CD31 was assessed by qPCR, Western blot, IHC, and ELISA. Ectopic lesions showed significantly elevated TLR4/NF-κB/NLRP3/IL-1β signaling compared with eutopic and control endometrium (all *p* < 0.05). Peritoneal fluid IL-1β was increased in patients, indicating a localized pelvic inflammatory response. In vitro, TAK-242 suppressed LPS/ATP-induced NF-κB/NLRP3 activation, pyroptosis, and IL-1β secretion (*p* < 0.05). Furthermore, the NLRP3-specific inhibitor MCC950 confirmed the essential role of NLRP3 inflammasome activation in IL-1β maturation. In vivo, TLR4 deletion or TAK-242 treatment reduced lesion weight, PCNA proliferation, and CD31 microvessel density (all *p* < 0.05). TLR4 inhibition blocks NF-κB nuclear translocation and subsequent inflammasome activation, suggesting a potential role in attenuating inflammation and angiogenesis. The TLR4/NF-κB/NLRP3 axis may drive endometriosis progression by linking innate immunity, inflammasome activation, pyroptosis, with possible involvement in angiogenesis warranting further investigation. Pharmacological inhibition of TLR4 attenuates lesion growth, supporting TLR4 as a promising non-hormonal therapeutic target for endometriosis.

## 1. Introduction

Endometriosis (EMS) is a chronic estrogen-dependent inflammatory disorder characterized by the presence of endometrial-like tissue outside the uterine cavity, which elicits dysmenorrhea, chronic pelvic pain, and infertility [1]. Affecting approximately 10% of women of reproductive age, EMS exerts a profound adverse impact on quality of life and mental well-being [2]. Its prevalence reaches 40–60% in women with severe menstrual disorders and chronic pelvic pain and affects nearly 50% of infertile women [3]. Mounting evidence confirms that chronic inflammation and immune dysregulation are key pathogenic mechanisms underlying EMS development and progression [3].

Damage-associated molecular patterns (DAMPs) act as critical stimuli triggering innate immune activation, thereby inducing non-infectious inflammatory responses [4]. Endometriotic lesions are hypothesized to undergo a cycle of repeated tissue injury and repair (ReTIAR) [5]. Notably, damaged cells secrete DAMPs, which—similar to pathogen-associated molecular patterns (PAMPs)—can activate the innate immune system [6]. Both PAMPs and DAMPs engage pattern recognition receptors, particularly toll-like receptors (TLRs), which have been implicated in EMS pathogenesis [7].

TLRs are transmembrane proteins comprising three domains: an extracellular domain responsible for recognizing PAMPs and DAMPs, a transmembrane domain, and an intracellular domain that mediates downstream signal transduction [8]. TLRs expressed in the endometrium drive the production of pro-inflammatory cytokines, including interleukin-1β (IL-1β) [9]. Previous studies have shown that TLR4 expression is significantly elevated in the endometrium of EMS patients compared with healthy controls [10,11], and our prior work further demonstrated TLR4 upregulation in endometriotic lesions [12]. TLR4 activation triggers a signaling cascade encompassing the nuclear factor κB (NF-κB) pathway, which drives transcription of genes linked to inflammation [13].

Accumulating evidence indicates that inflammasomes participate in inflammation-associated intracellular signaling cascades [14]. Inflammasomes sense diverse PAMPs and DAMPs, with nucleotide-binding oligomerization domain-like receptors (NLRs) serving as key intracellular sensors. NLRP3, a prototypical NLR family member, undergoes oligomerization upon activation, recruiting the adaptor apoptosis-associated speck-like protein containing a caspase recruitment domain (ASC) and caspase-1 protease, thereby triggering caspase-1 activation. Activated caspase-1 drives the maturation of IL-1β [9]. High mobility group box 1 (HMGB1), a prototypical DAMP, functions as an intranuclear DNA-binding protein that regulates DNA stabilization and transcription [15]. Upon release into the extracellular space during cell death, HMGB1 acts as an endogenous ligand activating inflammatory signaling pathways through its main receptor TLR4 [16]. Based on these evidences and our prior findings [12,17], we hypothesize that the TLR4/NF-κB/NLRP3 signaling axis plays a critical role in the pathogenesis and progression of endometriosis.

To verify this hypothesis, we first evaluated the activation status of the TLR4/NF-κB/NLRP3 signaling axis in human endometriotic lesions and the secretion levels of IL-1β in peritoneal fluid and serum of endometriosis patients. We further validated the role of TLR4/NF-κB/NLRP3-mediated inflammation in driving EMS development using EMS mouse models and in vitro cell experiments. We showed that EMS involved activation of the TLR4/NF-κB/NLRP3 inflammasome axis and that depletion and pharmacological inhibition of TLR4 led to a marked reduction in endometriotic lesion formation. Taken together, these findings not only advance our understanding of EMS pathophysiology but also facilitate the identification of novel therapeutic targets for this disease.

## 2. Results

### 2.1. Aberrant Activation of the TLR4/NF-κB/NLRP3 Pathway in Endometriotic Lesions with Concomitant Elevation of IL-1β Levels

**Figure 1:**
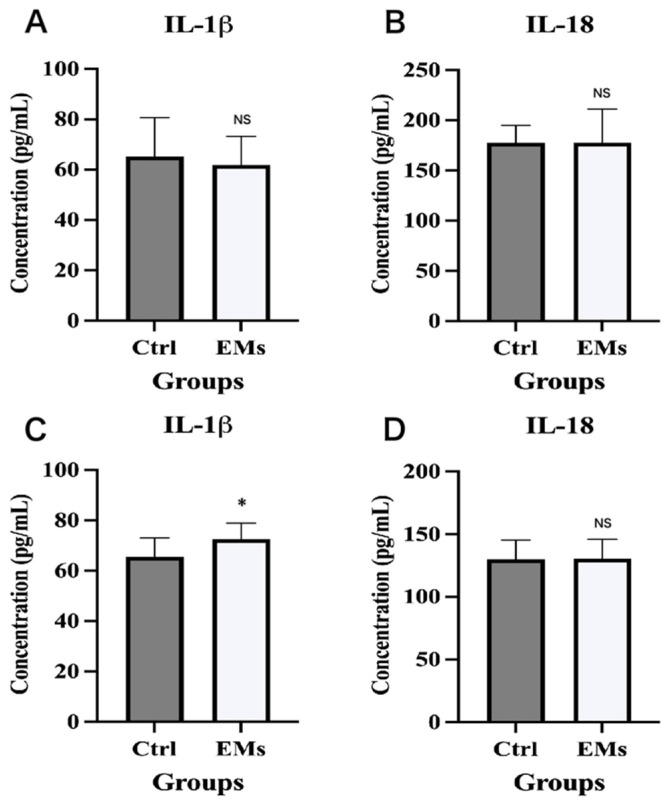
Peritoneal fluid IL-1β levels are elevated in endometriosis patients. (**A**,**B**) Levels of IL-1β and IL-18 in serum from EMS patients and non-EMS patients; (**C**,**D**) Levels of IL-1β and IL-18 in peritoneal fluid from EMS patients and non-EMS patients. * *p* < 0.05, NS, not statistically significant.

We measured IL-1β and IL-18 concentrations in serum and peritoneal fluid from 15 endometriosis patients and 15 non-endometriosis controls. There were no statistically significant differences in baseline characteristics between the two groups, with the exception of dysmenorrhea (*p* < 0.0001, Table 1). Results showed that compared with non-endometriosis controls, peritoneal fluid IL-1β levels were significantly elevated in endometriosis patients (*p* = 0.035, Figure 1C), whereas serum IL-1β concentrations exhibited no significant difference between the two groups (*p* = 0.497, Figure 1A). In addition, serum and peritoneal fluid IL-18 levels showed no significant intergroup differences (all *p* > 0.05, Figure 1B,D). These findings indicated that IL-1β, but not IL-18, was specifically elevated in the peritoneal microenvironment, suggesting a local inflammatory response in endometriosis rather than a systemic cytokine disturbance.

**Figure 2:**
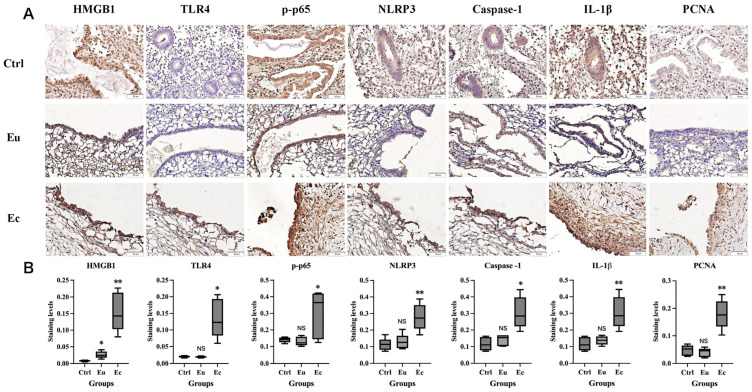
TLR4/NF-κB/NLRP3 pathway is aberrantly activated in endometriotic lesions. (**A**) Representative IHC staining microphotographs of HMGB1, TLR4, p-p65, NLRP3, caspase-1, IL-1β, and PCNA (scale bar = 50 μm; Magnification: 400×). Ctrl: normal endometrial tissue from women without EMS; Eu: eutopic endometrium from women with EMS; Ec: ectopic endometrium from women with EMS. Representative images from proliferative phase; (**B**) Box plots of HMGB1, TLR4, p-p65, NLRP3, caspase-1, IL-1β, and PCNA staining levels in the epithelial component among different groups. * *p* < 0.05, ** *p* < 0.01, NS, not statistically significant.

IHC staining was used to detect the expression of TLR4, p-p65, NLRP3, Caspase-1, IL-1β, and PCNA in 5 normal endometrial tissues (Ctrl), and paired ectopic (Ec) and eutopic (Eu) endometrial tissues from 5 ovarian endometriosis patients. Other characteristics were comparable between the two groups, except for dysmenorrhea (*p* < 0.0001, Table 2). As shown in Figure 2A, in ectopic tissues, TLR4, NLRP3, Caspase-1, and IL-1β exhibited strong cytoplasmic expression in epithelial cells, with weak cytoplasmic expression in stromal cells. P-p65 was expressed in both the cytoplasm and nucleus of epithelial and stromal cells, with higher expression levels in epithelial cells. PCNA showed nuclear localization in epithelial and stromal cells, with predominant expression in epithelial cells. Notably, the expression levels of all the above markers were upregulated in ectopic tissues compared with eutopic and normal endometria (all *p* < 0.05), with no significant differences observed between normal and eutopic endometrial tissues (all *p* > 0.05) (Figure 2B). Cycle phase distribution was comparable between groups (Table 2, *p* = 0.500). Taken together, these results confirmed the specific activation of the TLR4/NF-κB/NLRP3 inflammasome axis and the upregulation of downstream pro-inflammatory cytokine IL-1β in ectopic lesions, implicating this pathway in the pathogenesis of local inflammation in endometriosis.

### 2.2. Genetic Deletion or Pharmacological Inhibition of TLR4 with TAK-242 Suppresses Endometriotic Lesion Formation

**Figure 3:**
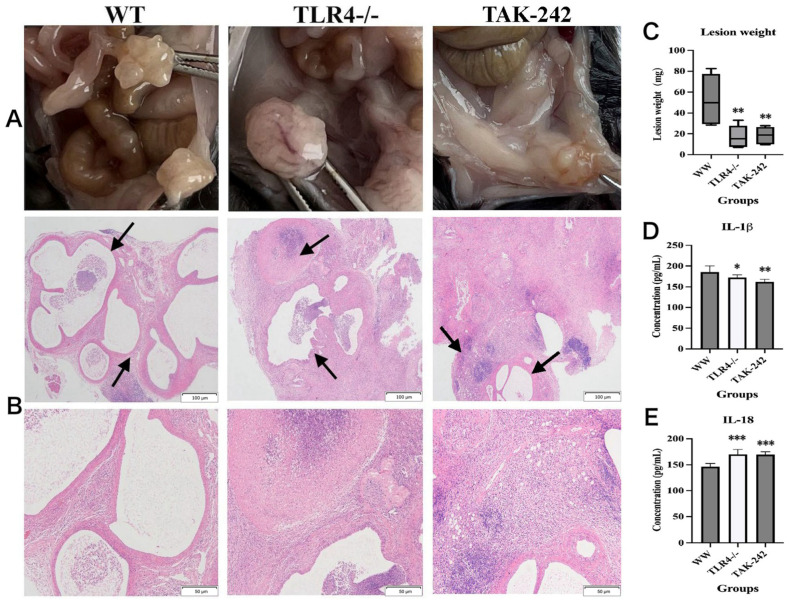
TLR4 ablation or TAK-242 therapy reduces endometriotic lesion burden. (**A**) Gross morphology of EMS lesion formation in different modeling groups; (**B**) EMS lesion formation in different modeling groups under H&E staining (scale bar = 100 μm or 50 μm; Magnification: 200× or 400×). Black arrows indicate endometriotic lesions and necrotic tissue; (**C**) Lesion weight of EMS in different modeling groups; (**D**,**E**) Serum levels of IL-1β and IL-18 in different modeling groups. * *p* < 0.05; ** *p* < 0.01; *** *p* < 0.001.

To validate the clinical findings in endometriosis patients, we performed complementary animal experiments, with no mortality occurring during the study period. The TLR4 knockout status of TLR4^−^/^−^ mice was confirmed by genotyping and IHC (Additional file 1: Figure S1). Gross morphological examination showed that wild-type (WT) mice developed multilocular translucent cystic lesions; TLR4^−^/^−^ mice formed encapsulated masses; and TAK-242-treated mice presented with small translucent cystic lesions on nodule surfaces (Figure 3A). Concordantly, endometriotic lesion weight was significantly lower in the TLR4^−^/^−^ and TAK-242-treated groups than in the WT group (*p* = 0.006 and *p* = 0.007, respectively) (Figure 3C). Hematoxylin– eosin (H&E) staining demonstrated classic endometriotic lesions in WT mice, whereas only sparse foci of endometriotic lesions were identified amid the necrotic tissue of TLR4^−^/^−^ and TAK-242-treated mice (Figure 3B). Thus, these data demonstrate that inactivation of TLR4 suppresses endometriotic lesion growth and survival, confirming the critical role of the TLR4 signaling pathway in endometriosis pathogenesis.

### 2.3. TLR4 Deficiency or TAK-242-Mediated TLR4 Inhibition Attenuates NF-κB/NLRP3 Pathway Activation and Impairs Neovascularization and Cell Proliferation

**Figure 4:**
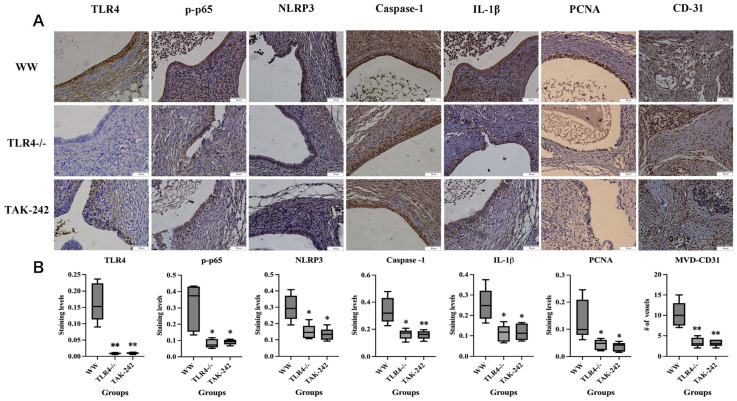
TLR4 ablation or TAK-242 treatment blunted NF-κB/NLRP3 pathway activation, suppressed neovascularization and proliferation. (**A**) Representative IHC staining microphotographs of TLR4, p-p65, NLRP3, caspase-1, IL-1β, PCNA, and CD31 (scale bar = 50 μm; Magnification: 400×); (**B**) Box plots of TLR4, p-p65, NLRP3, caspase-1, IL-1β, PCNA, and CD31 staining levels in the epithelial component among different groups. * *p* < 0.05, ** *p* < 0.01.

We next measured serum IL-1β and IL-18 levels in all mice, and found that serum IL-1β levels were significantly reduced in TLR4^−^/^−^ and TAK-242-treated mice compared with WT mice (*p* = 0.018 and *p* = 0.001, respectively; Figure 3D), whereas serum IL-18 levels were markedly elevated (all *p* < 0.001; Figure 3E). To confirm these effects at the tissue level, we then evaluated the immunoreactivity of TLR4, p-p65, NLRP3, Caspase-1, IL-1β, PCNA, and CD31 in lesions. As shown in Figure 4A, the expression patterns of these markers in WT lesions were consistent with that observed in human ectopic endometrial tissues, whereas their expression levels were significantly decreased in both the TLR4^−^/^−^ and TAK-242-treated mice endometriotic lesions (all *p* < 0.05; Figure 4B). Moreover, CD31-labeled neovascular density in lesions of the WT group was significantly higher than that in the other two groups (all *p* < 0.01; Figure 4B).

### 2.4. TAK-242 Inhibits TLR4/NF-κB/NLRP3 Activation and IL-1β Secretion in 11Z Cells

**Figure 5:**
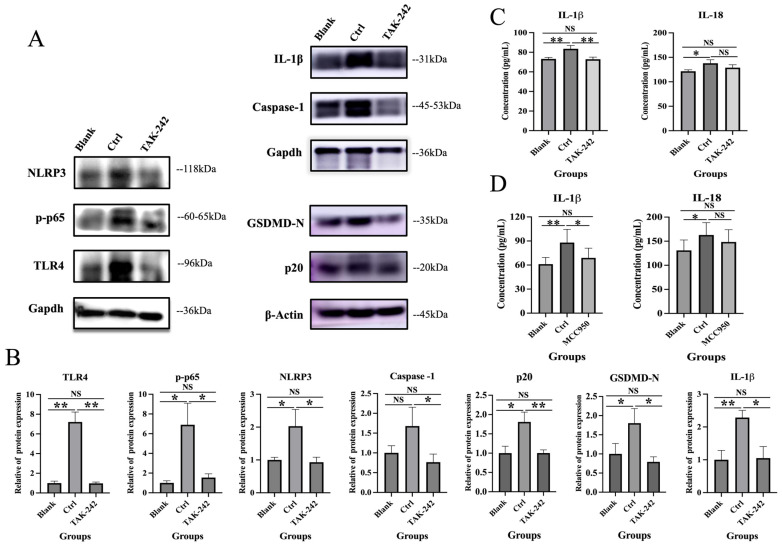
Differences in TLR4, p-p65, NLRP3, caspase-1, cleaved caspase-1 (p20), GSDMD-N, and IL-1β protein levels, and IL-1β and IL-18 concentrations in 11Z cell supernatant among different treatment groups. (**A**,**B**) Differences in protein levels of TLR4, p-p65, NLRP3, caspase-1, cleaved caspase-1 (p20), GSDMD-N, and IL-1β in 11Z cells across different treatment groups; (**C**) Concentrations of IL-1β and IL-18 in the supernatant of 11Z cells with TLR4 inhibitor (TAK-242) treatment; (**D**) Concentrations of IL-1β and IL-18 in the supernatant of 11Z cells with NLRP3 inhibitor (MCC950) treatment. * *p* < 0.05, ** *p* < 0.01, NS, not statistically significant.

**Figure 6:**
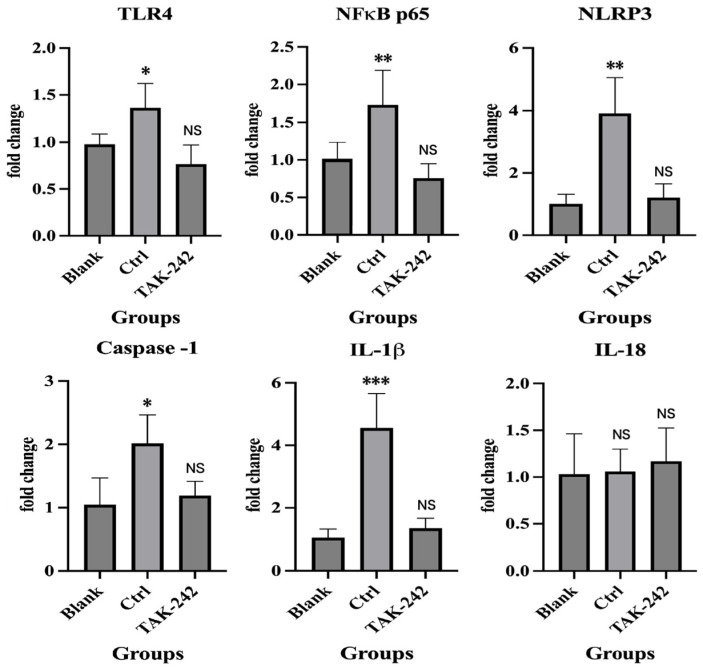
Differences in mRNA levels of TLR4, p-p65, NLRP3, caspase-1, IL-1β, and IL-18 in 11Z cells across different treatment groups. * *p* < 0.05, ** *p* < 0.01, *** *p* < 0.001, NS, not statistically significant.

To further verify the role of the TLR4/NF-κB/NLRP3 pathway in endometriosis, we conducted additional in vitro experiments. Results showed that compared with the Ctrl group, TAK-242 significantly reduced IL-1β concentrations in cell supernatants to levels comparable to the Blank group (*p* = 0.003 vs. Ctrl, *p* = 0.853 vs. Blank; Figure 5C). qRT-PCR and Western blot (WB) analyses confirmed that TAK-242 treatment markedly down-regulated the mRNA and protein expression of TLR4, p-p65, NLRP3, Caspase-1, cleaved caspase-1 (p20), GSDMD-N, and IL-1β in 11Z cells, with levels restored to those of the Blank group (all *p* < 0.05 vs. Ctrl, all *p* > 0.05 vs. Blank; Figure 5A,B and Figure 6). Notably, TAK-242 treatment had no significant effect on IL-18 secretion or mRNA expression in 11Z cells, as IL-18 levels remained unchanged across all treatment groups (all *p* > 0.05; Figure 5C and Figure 6). Furthermore, pretreatment with the specific NLRP3 inhibitor MCC950 (0.1 μM) significantly suppressed IL-1β secretion (*p* < 0.05; Figure 5D), but did not affect IL-18 expression. These results further validate the essential role of NLRP3 inflammasome activation. In summary, these in vitro findings demonstrated that TAK-242 specifically inhibited TLR4/NF-κB/NLRP3 pathway activation and IL-1β production without affecting IL-18 expression, which were consistent with our in vivo observations.

## 3. Discussion

Endometriosis is increasingly recognized as a chronic inflammatory disorder underpinned by immune dysregulation within the pelvic microenvironment [3]. In the present study, we integrate data from human specimens, murine EMS models, and endometriotic epithelial cell experiments to present convergent evidence demonstrating that aberrant activation of the TLR4/NF-κB/NLRP3 axis augments IL-1β production, promotes pyroptosis via cleaved caspase-1 (p20) and GSDMD-N, facilitates neovascularization, and enhances cell proliferation, ultimately fueling the formation and progression of EMS.

As a key pro-inflammatory cytokine, IL-1β plays a central role in EMS pathogenesis. Consistent with prior findings [18,19], our study confirms that IL-1β levels are markedly elevated in the peritoneal fluid but unaltered in the serum of EMS patients—highlighting a compartmentalized inflammatory response confined to the pelvic microenvironment. Notably, in contrast to IL-1β, IL-18 levels exhibited no significant differences between EMS patients and healthy controls in either serum or peritoneal fluid, an observation consistent with previous work by Fairbanks [20]. This divergence likely reflects distinct regulatory mechanisms governing pro-IL-18 bioavailability, inflammasome-dependent processing, or cell-type-specific production within the endometriotic niche. Beyond the canonical caspase-1 pathway, endometrial cells can also activate non-canonical inflammasome signaling (e.g., caspase-4/5 in humans), which fine-tunes IL-1 family cytokine outputs in a stimulus-dependent manner [21]. In contrast, our murine model showed elevated serum IL-18 in TLR4^−^/^−^ and TAK-242-treated mice (Figure 3E). This species discrepancy may reflect differential regulation of IL-18 binding protein (IL-18BP), distinct inflammasome activation kinetics between acute and chronic settings, or compensatory pathways upon TLR4 deficiency. However, conflicting reports have shown that both IL-1β and IL-18 are significantly elevated in both serum and peritoneal fluid of EMS patients [22,23]. This discrepancy is likely attributable to the heterogeneous phenotypes of EMS, as recent research has demonstrated that peritoneal and ovarian EMS subtypes harbor distinct immune microenvironments, with differential expression of immune-associated molecules [24]. Thus, delineating inflammasome signaling networks by cell type (epithelial vs. stromal cells) and lesion subtype (ovarian vs. peritoneal) will be essential for advancing the therapeutic translation of these findings.

Prior studies have demonstrated that the expression level of IL-1β in ectopic lesions of deep endometriosis (DE) is significantly higher than that in eutopic endometrial tissue during both the proliferative and secretory phases [25]. IL-1β is also reported to promote the proliferation of endometrial stromal cells [26]; consistent with these findings, our study further shows that the expression of PCNA—a canonical marker of cell proliferation—increases with the elevation of IL-1β expression levels in endometriotic lesions. Relative to both eutopic and normal endometrial tissues, ectopic lesions displayed marked upregulation of TLR4, p-p65, NLRP3, caspase-1, IL-1β, and PCNA. Our previously published data have confirmed that plasma HMGB1 levels are elevated in both EMS patients and animal models, and the HMGB1/TLR4 pathway is abnormally activated in ectopic lesions [12]. HMGB1 functions as an intranuclear DNA-binding protein in mammalian cells, regulating DNA stabilization and transcriptional processes [15]. Upon release into the extracellular space during cell death, HMGB1 acts as an endogenous ligand that activates inflammatory signaling pathways and is recognized as a prototypical DAMP which can bind to its main receptor TLR4 [16]. Thus, these clinical findings are consistent with previous reports that link sterile inflammation and DAMP signaling to sustained inflammatory activation in EMS [4,6,7]. Taken together, these data corroborate and extend our prior work by establishing the HMGB1/TLR4/NF-κB/NLRP3 signaling axis as a pivotal inflammatory driver in endometriotic tissues and cells [13,17].

Angiogenesis is indispensable for the survival and expansion of endometriotic lesions. NF-κB signaling is widely recognized as a key driver of inflammation, invasion, angiogenesis, and proliferation in EMS, with constitutive activation of this pathway well-documented in both endometriotic lesions and peritoneal immune cells [27]. IL-1β can reinforce this signaling circuitry by amplifying inflammatory cytokine networks and inducing angiogenic mediators (e.g., the VEGF family) in endometriotic cells as well as associated stromal and immune compartments [28]. Contemporary reviews highlight that angiogenic programming in endometriosis integrates hormonal cues, inflammatory cytokines, and immune cell recruitment, with NF-κB serving as a central transcriptional hub [29,30]. In this context, our observation that ectopic lesions exhibit elevated PCNA expression concurrent with upregulated inflammatory pathway markers suggests that the TLR4/NF-κB/NLRP3/IL-1β axis may coordinately promote cellular proliferation and microenvironmental remodeling in EMS. While we did not directly quantify angiogenic cytokines (e.g., VEGF-A, VEGF-C) in the current dataset, prior studies have shown that IL-1β can upregulate lymphangiogenic and angiogenic mediators in EMS models, underscoring a plausible mechanistic link between inflammasome activation and vascular remodeling [31]. Given the reported role of NF-κB and IL-1β in angiogenic processes, we assessed neovascularization in ectopic lesions and detected increased neovascular density (CD31 staining) in these lesions.

TLR4 activation triggers NF-κB-dependent transcriptional “priming” of inflammasome components and pro-inflammatory cytokines—a process essential for the subsequent assembly of the NLRP3 inflammasome and caspase-1-mediated IL-1β maturation [8,14]. Consistent with this canonical two-step model of inflammasome activation, our IHC data revealed concomitant upregulation of p-p65, NLRP3, caspase-1, and IL-1β in ectopic lesions both in human and mouse samples. We further identify that targeting TLR4 can abrogate downstream inflammatory amplification cascades and lesion-promoting biological processes. In vitro, LPS/ATP-stimulated 11Z cells treated with the selective TLR4 inhibitor TAK-242 exhibited marked reductions in IL-1β secretion, alongside downregulated expression of TLR4, p-p65, NLRP3, caspase-1, cleaved caspase-1 (p20), GSDMD-N, and IL-1β. Furthermore, the specific NLRP3 inhibitor MCC950 recapitulated these inhibitory effects, confirming the essential role of NLRP3 inflammasome activation and pyroptosis in this process. These results provide compelling functional evidence supporting the notion that TLR4 acts upstream of NF-κB/NLRP3 activation in EMS, which aligns with extensive published evidence documenting TLR4-dependent inflammatory cytokine induction in reproductive tissues [9,10]. Importantly, using a well-established murine EMS model, we implemented dual interventions—genetic ablation of TLR4 and pharmacologic inhibition via TAK-242—an approach that bolsters the causal link between TLR4 and pathway activity, validating it as a key node amenable to targeted modulation. Our results further demonstrate that TLR4 inhibition or deficiency markedly attenuates lesion formation, while concurrently suppressing pathway activation, cellular proliferation, and neovascularization (as assessed by PCNA and CD31 expression). Collectively, these findings establish that TLR4 blockade constitutes a mechanism-driven therapeutic strategy that concurrently inhibits the maturation of inflammasome-associated cytokines and the downstream tissue remodeling processes that underpin lesion persistence and progression.

At the preclinical level, this study provides a robust theoretical basis for the growing consensus that “the pathophysiological mechanism of EMS entails the integration of inflammation, neuroangiogenesis, and wound healing processes” [3,5,28]. As a potent driver of secondary cytokine networks, IL-1β regulates angiogenesis and neurotrophic pathways associated with pain pathogenesis and lesion persistence [28,32]. In our experimental EMS models, attenuated IL-1β signaling correlates closely with the downregulated expression of angiogenesis and proliferation markers, indicating that the TLR4/NF-κB/NLRP3 axis may act as a central “hub” mediating crosstalk between innate immune sensing, lesion vascularization, and progressive growth. At the clinical level, current first-line therapies for EMS are predominantly hormonal or surgical, which are often plagued by high recurrence rates and notable adverse effects [3,33]. In contrast, a non-hormonal, inflammation-targeted therapeutic strategy—potentially in combination with standard regimens—holds considerable promise for expanding the therapeutic armamentarium, particularly for patients with prominent inflammation-driven phenotypes. Nevertheless, systemic TLR4 blockade warrants cautious implementation, given the pivotal role of TLR4 in mediating host immune defense [8]. TAK-242 has undergone clinical evaluation in sepsis and shock; while its tolerability profile was generally favorable, efficacy signals were limited and cytokine suppression proved suboptimal in that clinical setting [34]. This clinical history does not negate the agent’s potential utility in endometriosis, but it underscores the critical importance of optimized dosing, precision patient stratification, and potentially localized delivery approaches (e.g., intraperitoneal formulations) to maximize pelvic therapeutic benefits while minimizing systemic immunomodulatory side effects.

Given that the inflammatory effects of this pathway converge on IL-1β, another viable translational approach lies in targeting the downstream IL-1 signaling axis. A recent randomized crossover pilot study of the IL-1 receptor antagonist anakinra (Kineret^®^) demonstrated improved endometriosis-related quality of life, reduced pain, and exploratory inflammatory biomarker profiling—validating the clinical feasibility of IL-1-targeted non-hormonal interventions [35]. Additionally, direct NLRP3 inhibition represents a feasible strategy: MCC950 attenuated IL-1β secretion in primary human endometrial stromal cells and exerted beneficial effects in experimental models of ovarian EMS, supporting inflammasome inhibition as a complementary or alternative approach to IL-1 axis targeting [21]. Collectively, these data delineate a clear therapeutically actionable signaling continuum: TLR4 (upstream sensing) → NF-κB (inflammasome priming) → NLRP3/caspase (cytokine processing) → IL-1 signaling (effector responses). This continuum enables stratified intervention based on patient phenotype and lesion-specific biological characteristics.

Several limitations should be acknowledged. First, the sample size was relatively small (15 per group), and only ovarian EMS was included; larger stratified studies are needed. Second, the LPS/ATP in vitro model does not fully recapitulate the DAMP-rich peritoneal microenvironment of EMS. Third, separate quantification of nuclear and cytoplasmic p-p65 was difficult in some lesions due to unclear nuclear–cytoplasmic boundaries; we therefore assessed overall immunoreactivity to reflect NF-κB activation. Future studies using HMGB1 or patient-derived peritoneal fluid, combined with high-resolution imaging for p-p65 translocation, are warranted to further validate the TLR4/NLRP3/IL-1β axis.

## 4. Materials and Methods

### 4.1. Reagents

Anti-human/mouse primary antibodies against HMGB1, TLR4, phosphorylated NF-κB p65 (p-p65), caspase-1, PCNA, and CD31 were purchased from Abcam (Cambridge, UK). The cleaved caspase-1 (p20) and IL-1β-targeting antibodies were obtained from Affinity Biosciences (Changzhou, China), whereas anti-human/mouse NLRP3, and GSDMD-N antibodies were purchased from HUABIO (Hangzhou, China). The details of all primary antibodies used in this study are summarized in Table 3. Human- and mouse-specific IL-1β and IL-18 ELISA kits were procured from Jiangsu Meimian Industrial Co., Ltd. (Yancheng, China). The TLR4 inhibitor TAK-242 and NLRP3 inhibitor MCC950 were purchased from Selleck Chemicals (Houston, TX, USA), and both LPS and ATP were obtained from Beyotime Biotechnology Inc (Beijing, China).

### 4.2. Patients and Specimen Collection

Fifteen premenopausal patients with ovarian endometriosis (OE), confirmed by laparoscopy and histopathology, were assigned to the EMS group; 15 age-matched premenopausal non-endometriosis patients served as the Ctrl group. Baseline characteristics of all participants are presented in Table 1. Preoperative peripheral blood samples from all participants were centrifuged to isolate serum and stored at −80 °C for subsequent assays. Intraoperatively, ectopic and eutopic endometrial tissues were harvested from the EMS group, and normal endometrial tissues from the Ctrl group; all tissues were fixed in 4% paraformaldehyde and paraffin-embedded for downstream experiments. For IHC analysis, paired ectopic and eutopic endometrial tissues from 5 ovarian endometriosis patients and normal endometrial tissues from 5 controls were used; their characteristics are summarized in Table 2. Peritoneal fluid from both groups was centrifuged to collect supernatant and preserved at −80 °C for later use.

This study was conducted in strict accordance with the ethical principles of the Declaration of Helsinki. Written informed consent was obtained from all participants prior to enrollment. The study protocol was reviewed and approved by the Ethics Committee of Obstetrics and Gynecology Hospital, Fudan University (Approval No.: 2023-112, Approval Date: 26 December 2023). No patient had received hormonal therapy for at least 3 months prior to surgery.

### 4.3. Animals and Induction of Endometriosis

C57BL/6 mice were purchased from Shanghai Slake Experimental Animal Co., Ltd. (Shanghai, China), and TLR4-deficient (TLR4^−^/^−^) mice from GemPharmatech Co., Ltd. (Nanjing, China). All mice were acclimatized for 1 week before experimental treatments. A total of 24 female C57BL/6 mice (6–8 weeks old) and 12 female TLR4^−^/^−^ mice (6–8 weeks old) were housed in a specific pathogen-free (SPF) barrier facility under a 12 h light/dark cycle, with free access to water and standard chow. All animal experiments were approved by the Animal Ethics Committee of Shanghai University of Traditional Chinese Medicine (Approval No.: PZSHUTCM2301060001, Approval Date: 6 January 2023) and complied with the ARRIVE guidelines and international laboratory animal care standards.

Of the 24 C57BL/6 mice, 8 were randomly selected as uterine tissue fragment donors, with the remaining 16 serving as recipients. For the 12 TLR4^−^/^−^ mice, 4 were randomly chosen as donors and the other 8 as recipients. Following 1 week of acclimation, all donor mice received intramuscular injections of 100 mg/kg estradiol benzoate (Hangzhou Animal Pharmaceutical Factory, Hangzhou, China) twice weekly.

Following 1 week of estrogen injections in all donors, 16 C57BL/6 recipients were randomly split into two groups (Ctrl, *n* = 8; TAK-242, *n* = 8). One day before model induction, the TAK-242 group received 3 mg/kg TAK-242 intraperitoneally, while the Ctrl and TLR4^−^/^−^ groups received equal-volume vehicle intraperitoneally. The endometriosis model—first described by Somigliana E et al. [36] and previously used in our study [12] —was established via intraperitoneal injection of uterine tissue fragments. Specifically, donors were euthanized, uteri excised, rinsed twice with sterile normal saline, longitudinally incised, and cut into <1 mm fragments. Fragments were suspended in sterile normal saline and injected into recipients’ peritoneal cavities, with tissue from each donor equally allocated to two recipients. Post-induction, the TAK-242 group received 3 mg/kg TAK-242 intraperitoneally twice weekly; the Ctrl and TLR4^−^/^−^ groups received vehicle intraperitoneally on the same schedule.

Two weeks post-model induction, all mice were euthanized. Peripheral blood was collected, centrifuged to obtain serum, and stored at −80 °C for subsequent assays. Endometriotic lesions were harvested and fixed in 4% paraformaldehyde and paraffin-embedded for histological analysis. Mice were randomly assigned to groups using a random number table. Outcome assessments were performed blinded to group allocation.

### 4.4. Cell Culture and Treatment

The 11Z cell line (FH-H068; Shanghai Fuheng Biotechnology Co., Ltd., Shanghai, China) was thawed, subcultured for 3 passages, and used for experiments. The 11Z cell line was authenticated by STR profiling and tested negative for mycoplasma. Cells were cultured in DMEM/F12 supplemented with 10% fetal bovine serum (FBS) and 1% penicillin/streptomycin in a humidified 37 °C incubator with 5% CO_2_. After seeding into 6-well plates, cells were serum-starved overnight in serum-free medium and divided into Blank, Ctrl, TAK-242, and MCC950 groups. The TAK-242 group was pretreated with 3 μM TAK-242 for 2 h, and the MCC950 group was pretreated with 0.1 μM MCC950 for 1 h, whereas the other two groups received equal-volume vehicle. Medium was then replaced: Ctrl and TAK-242 groups were treated with 200 ng/mL LPS for 48 h, while the Blank group received vehicle. Following another medium change, all groups were treated with 3 mM ATP for 45 min. Cells and culture supernatants were harvested for subsequent assays.

### 4.5. Hematoxylin and Eosin Staining and Immunohistochemistry

4-μm-thick paraffin-embedded sections were dewaxed and rehydrated following standard protocols. Hematoxylin and eosin (H&E) staining was performed using an H&E Staining Kit (Sun Biotec, Shanghai, China). For immunohistochemistry (IHC), after dewaxing and rehydration, antigen retrieval was performed using citrate or EDTA buffer (pH 9.0). Following cooling to RT, sections were incubated with primary antibodies (optimal dilutions) at 4 °C overnight. Sections were then washed with PBS and incubated with HRP-conjugated secondary antibody (Sunpoly-HII, Wuhan, China) at RT for 1 h. Negative controls were performed by omitting the primary antibody or substituting with IgG isotype control. Positive controls were performed using manufacturer-recommended positive tissues. Representative negative and positive controls are shown in Additional file 2: Figure S2. Antigen–antibody complexes were visualized with 3,3’-diaminobenzidine (DAB) for 30s–3min (optimized for microscopic observation), followed by hematoxylin counterstaining for 1 min and mounting with neutral balsam.

Images were captured using a microscope (Olympus BX53) equipped with a digital camera (Olympus DP73; Olympus Corporation, Tokyo, Japan). Four random fields per sample were imaged at 400× magnification, and mean optical density (MOD) was quantified via Image-Pro Plus 6.0 (Media Cybernetics, Inc., Rockville, MD, USA).

### 4.6. Enzyme-Linked Immunosorbent Assay

IL-1β and IL-18 levels in human and mouse serum and peritoneal fluid were quantified using commercial ELISA kits (Jiangsu Meimian Industrial Co., Ltd., Yancheng, China) according to the manufacturer’s instructions. Absorbance was measured at 450 nm, and standard curves were generated to calculate target analyte concentrations.

### 4.7. Quantitative Real-Time PCR

Total RNA was isolated from samples using TRIzol reagent (Invitrogen, Carlsbad, CA, USA) and reverse-transcribed to first-strand cDNA. Only RNA with OD_260_/OD_280_ ratio 1.8–2.0 was used to ensure purity. Primers were designed with Primer Premier software (Premier Biosoft, Palo Alto, CA, USA), and sequences are listed in Table 4. qRT-PCR was performed with SYBR Green I Master Mix (Takara, Dalian, China), using GAPDH as the reference gene for normalization. Relative expression of target genes was calculated via the 2^−ΔΔCt^ method.

### 4.8. Western Blotting

Intracellular proteins were extracted using RIPA lysis buffer (with protease and phosphatase inhibitors) and quantified by BCA protein assay. After SDS-PAGE, proteins were transferred to polyvinylidene fluoride (PVDF) membranes. Membranes were incubated with primary antibodies at 4 °C overnight, then with HRP-conjugated secondary antibodies for 1h at RT. Relative protein expression was normalized to GAPDH (loading control). Chemiluminescence signals were detected with an ECL kit, and band intensities were quantified using ImageJ 1.53 (National Institutes of Health, Bethesda, MD, USA).

### 4.9. Statistical Analysis

All statistical analyses were performed and graphs were plotted using GraphPad Prism 10.0 (GraphPad Software, San Diego, CA, USA). Data were presented as mean ± standard deviation (SD). Normality was assessed using the Shapiro–Wilk test, and homogeneity of variance was verified using Levene’s test. For data that passed both assumptions, parametric tests (Student’s t-test for two-group comparisons, one-way ANOVA with Tukey’s post hoc test for multi-group comparisons) were applied. For data that did not meet the assumption of equal variance (peripheral blood IL-18), Welch’s t-test was used. For multiple comparisons across multiple independent endpoints, Bonferroni correction was applied. Differences were considered statistically significant at *p* < 0.05. A priori power analysis using G * Power 3.1 (Universität Düsseldorf, Düsseldorf, Germany) estimated that 11 patients per group were required to detect changes in the primary biomarker (α = 0.05, power = 0.8, Cohen’s d = 1.29 based on pilot data). Our study included 15 per group. Biological replicates were independent patient samples or independently cultured cell passages (*n* ≥ 3). Technical replicates were repeated measurements from the same biological sample (e.g., duplicate ELISA wells, triplicate qPCR reactions).

## 5. Conclusions

In summary, our findings delineate a mechanistic model in which activation of TLR4/NF-κB signaling primes and subsequently activates the NLRP3 inflammasome, promotes pyroptosis by inducing cleaved caspase-1 (p20) and GSDMD-N, and facilitates caspase-1-mediated IL-1β maturation. This cascade thereby fuels endometriotic lesion progression by promoting local inflammation and enhancing cellular proliferation. Accordingly, targeting TLR4 represents a mechanism-driven therapeutic strategy with substantial potential for the development of non-hormonal treatments tailored to the inflammatory phenotype of EMS.

## Figures and Tables

**Table 1:** Baseline characteristics of all participants (*n* = 30).

Variable	Control (*n* = 15)	EMS (*n* = 15)	*p* Value
Age (years)	36.2 ± 5.44	35.8 ± 8.41	0.878
Gravidity	1.8 ± 1.45	2.0 ± 2.23	0.835
Parity	0.6 ± 0.53	0.8 ± 0.42	0.762
Menstrual phase			0.536
Proliferative	6 (40%)	8 (53%)	
Secretory	9 (60%)	7 (47%)	
Dysmenorrhea			0.000
Mild	N/A	2 (13%)	
Moderate	N/A	8 (53%)	
Severe	N/A	5 (33%)	
rASRM stage			N/A
Stage I	N/A	0	
Stage II	N/A	0	
Stage III	N/A	7 (47%)	
Stage IV	N/A	8 (53%)	
Other diseases			N/A
None	/	9 (60%)	
Teratoma	6 (40%)	/	
Adenomyosis	/	1 (6%)	
Leiomyoma	9 (60%)	5 (33%)	

Data are presented as mean ± SD or n (%). *p* < 0.0001 for dysmenorrhea between the two groups; other baseline characteristics were comparable (all *p* > 0.05). rASRM, revised American Society for Reproductive Medicine classification.

**Table 2:** Characteristics of participants for IHC analysis (*n* = 10).

Variable	Control (*n* = 5)	EMS (*n* = 5)	*p* Value
Age (years)	38.2 ± 6.30	44.0 ± 9.59	0.296
Gravidity	2.0 ± 1.41	2.2 ± 2.28	0.873
Parity	0.6 ± 0.55	0.8 ± 0.45	0.545
Menstrual phase			0.500
Proliferative	2 (40%)	3 (60%)	
Secretory	3 (60%)	2 (40%)	
Dysmenorrhea			0.000
Mild	N/A	1 (20%)	
Moderate	N/A	2 (40%)	
Severe	N/A	2 (40%)	
rASRM stage			N/A
Stage I	N/A	0	
Stage II	N/A	0	
Stage III	N/A	3 (60%)	
Stage IV	N/A	2 (40%)	
Other diseases			N/A
None	2 (40%)	4 (80%)	
Adenomyosis	1 (20%)	/	
Leiomyoma	2 (40%)	1 (20%)	

Data are presented as mean ± SD or n (%). *p* < 0.0001 for dysmenorrhea between the two groups; other baseline characteristics were comparable (all *p* > 0.05). rASRM, revised American Society for Reproductive Medicine classification.

**Table 3:** Primary antibodies used in this study.

Antibody Name	Catalog Number	Dilution IHC	Dilution WB	Company Name
GAPDH	#5174	/	1:1000	Cell Signaling Technology
HMGB1	Ab79823	/	1:1000	Abcam
TLR4	Ab22048	1:100	1:1000	Abcam
NF-κB p65 (phosphorylated)	Ab86299	1:300	1:2000	Abcam
NLRP3	ET1610-93	1:500	1:1000	HUABIO
Caspase-1	Ab179515	1:500	1:1000	Abcam
cleaved caspase-1 (p20)	AF4005	/	1:1000	Affinity
GSDMD-N	HA723254	/	1:1000	HUABIO
IL-1β	AF5103	1:300	1:1000	Affinity
PCNA	Ab29	1:200	/	Abcam
CD31	Ab124432	1:600	/	Abcam
β-Actin	#4967	/	1:1000	Cell Signaling Technology

All antibodies were used for immunohistochemistry (IHC) and Western blotting (WB) unless otherwise specified. Dilution ratios indicate working concentrations.

**Table 4:** Primer sequences for quantitative real-time PCR.

Gene	Forward (5′-3′)	Reverse (5′-3′)
TLR4	TCCATAAAAGCCGAAAGGTG	GATACCAGCACGACTGCTCA
NF-κB p65	ATGTGGAGATCATTGAGCAGC	CCTGGTCCTGTGTAGCCATT
NLRP3	CCACAAGATCGTGAGAAAACCC	CGGTCCTATGTGCTCGTCA
Caspase-1	TCCTCAGGCTCAGAAGGGAATGTC	GTGCGGCTTGACTTGTCCATTATTG
IL-1β	AGCTTGGTGATGTCTGGTCC	ACGCAGGACAGGTACAGATT
IL-18	AATGCACCCCGGACCATATTT	CCTGGGACACTTCTCTGAAAGA
Gapdh	GAAGGTGAAGGTCGGAGTC	GAAGATGGTGATGGGATTTC

All primers were designed for human genes. GAPDH was used as the internal reference gene. Forward, forward primer; Reverse, reverse primer.

## Data Availability

The datasets generated and analyzed during the current study are available from the corresponding authors upon reasonable request. The data include clinically annotated patient information and cannot be shared publicly to protect participant privacy. Additional quantitative data, image-derived experimental results, and raw data generated from in vitro and in vivo experiments are included in this published article and its supplementary information files.

## Supplementary Materials

The following supporting information can be downloaded at: https://www.mdpi.com/article/10.3390/ijms27094151/s1.

## Abbreviations

ASC	Apoptosis-associated speck-like protein containing a caspase recruitment domain
DAMP	Damage-associated molecular pattern
DMEM	Dulbecco’s Modified Eagle Medium
ELISA	Enzyme-linked immunosorbent assay
EMS	Endometriosis
FBS	Fetal bovine serum
GAPDH	Glyceraldehyde-3-phosphate dehydrogenase
H&E	Hematoxylin and eosin
HMGB1	High mobility group box 1
HRP	Horseradish peroxidase
IHC	Immunohistochemistry
IL-1β	Interleukin-1β
IL-18	Interleukin-18
LPS	Lipopolysaccharide
MOD	Mean optical density
NF-κB	Nuclear factor kappa-B
NLR	Nucleotide-binding oligomerization domain-like receptor
NLRP3	NLR family pyrin domain containing 3
OE	Ovarian endometriosis
PAMP	Pathogen-associated molecular pattern
PBS	Phosphate-buffered saline
PCNA	Proliferating cell nuclear antigen
PCR	Polymerase chain reaction
PVDF	Polyvinylidene fluoride
qRT-PCR	Quantitative real-time polymerase chain reaction
ReTIAR	Repeated tissue injury and repair
RIPA	Radioimmunoprecipitation assay
SEM	Standard error of the mean
SPF	Specific pathogen-free
TBST	Tris-buffered saline with Tween 20
TLR4	Toll-like receptor 4
VEGF	Vascular endothelial growth factor
WB	Western blot
WT	Wild-type
