# Supplementary figures and images for: Targeting TLR4 Attenuates Endometriosis Progression by Suppressing NF-κB/NLRP3 Inflammasome Activation and Angiogenesis
Source: Int J Mol Sci. 2026 May 6;27(9):4151. doi: 10.3390/ijms27094151 (PMC13164178; doi:10.3390/ijms27094151)

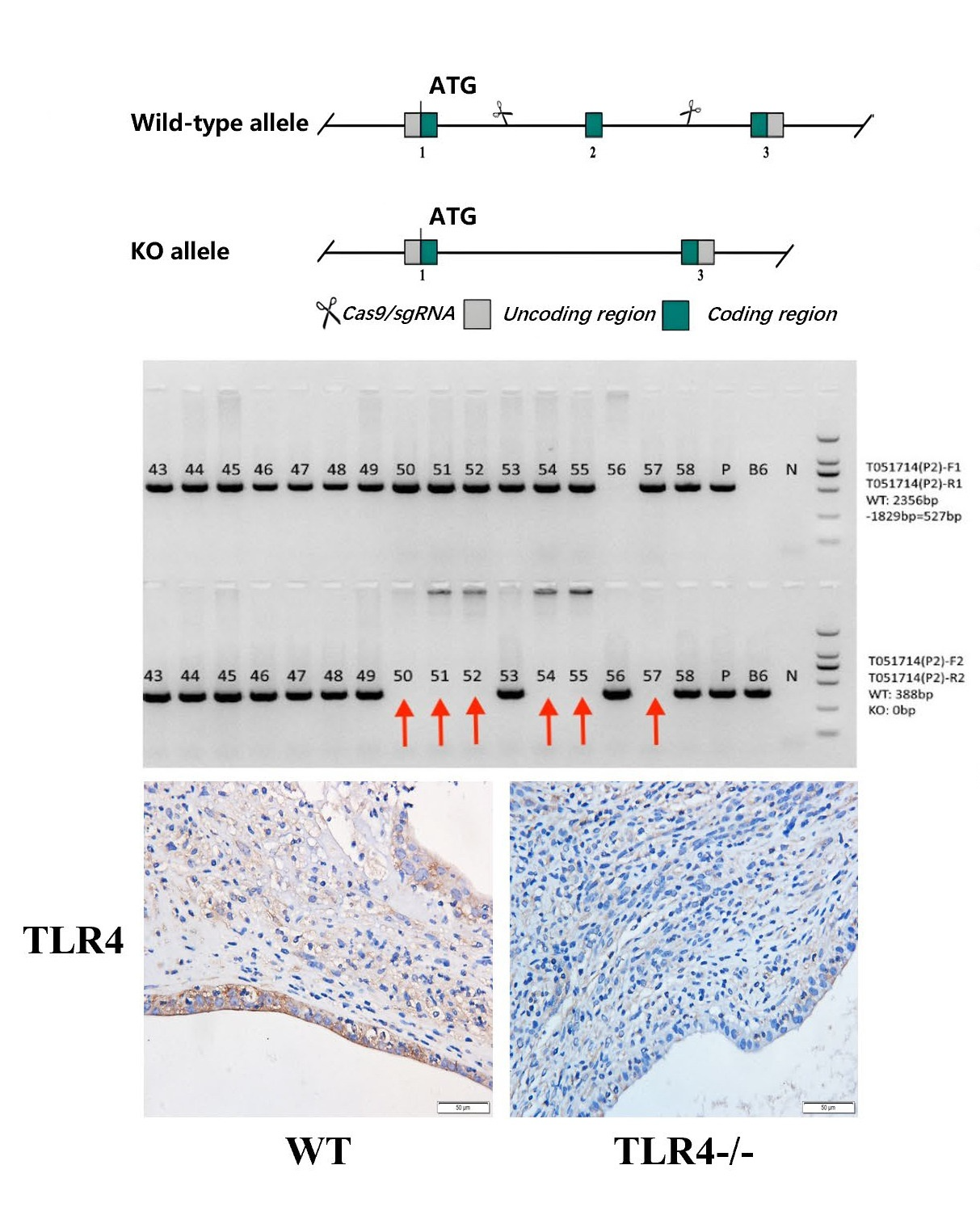

Supplement: Supplementary file 1 [file ijms-27-04151-s001.zip › Figure S1.tiff]

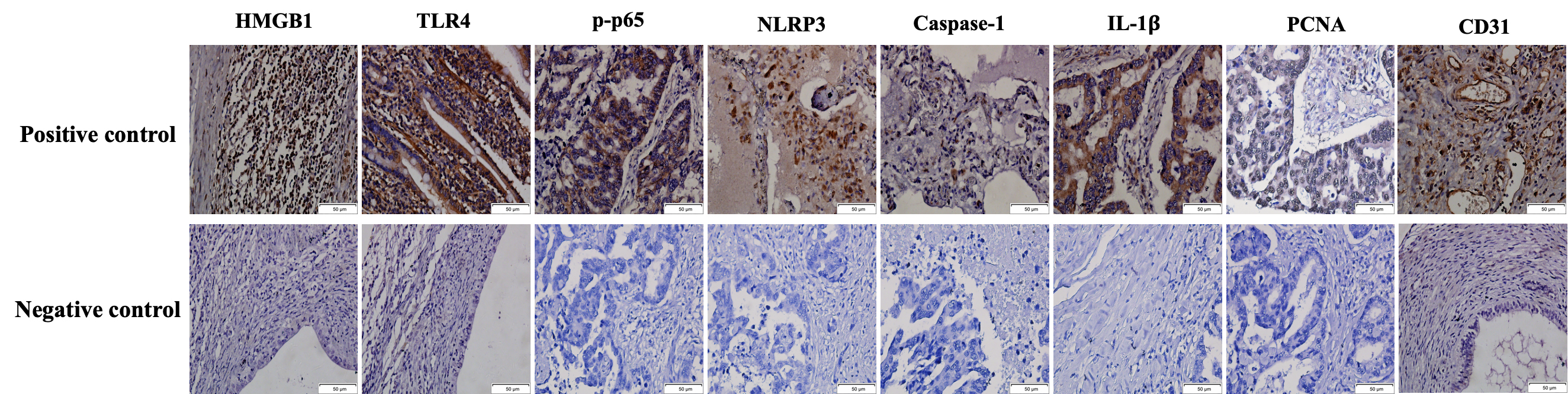

Supplement: Supplementary file 1 [file ijms-27-04151-s001.zip › Figure S2.tiff]
